# Survival outcomes, determinants, and hemodynamic trajectories with intravenous β_1_-selective blockade in septic shock complicated by tachyarrhythmia: a real-world MIMIC-IV cohort study

**DOI:** 10.3389/fphar.2026.1741601

**Published:** 2026-05-22

**Authors:** Guoxiang Zou, Xiujuan Chen, Xuanhui Chen, Shuai Huang, Feier Song, Bei Hu, Guangjian Liu, Xin Li, Huixian Li

**Affiliations:** 1 Department of Emergency Medicine, Guangdong Provincial People’s Hospital, Guangdong Academy of Medical Sciences, Southern Medical University, Guangzhou, China; 2 Medical Big Data Center, Guangdong Provincial People’s Hospital, Guangdong Academy of Medical Sciences, Southern Medical University, Guangzhou, China; 3 Guangdong Provincial Key Laboratory of Artificial Intelligence in Medical Image Analysis and Application, Guangzhou, China

**Keywords:** MIMIC-IV, mortality, predictor, septic shock, tachyarrhythmia, β1-selective blocker

## Abstract

**Background:**

Sepsis and septic shock impose severe cardiovascular stress characterized by sympathetic overactivation and detrimental tachycardia, which is strongly linked to poor outcomes. While ultra-short-acting β_1_-blockers have shown promise in controlled trials for heart rate (HR) control, a critical gap remains regarding the optimal β_1_-selective regimen for complex, multimorbid, vasopressor-dependent patients with septic shock complicated by tachyarrhythmia in real-world settings.

**Objective:**

To compare survival and hemodynamic trajectories associated with two intravenous β_1_-selective blockers—esmolol and metoprolol, when initiated during septic shock complicated by tachyarrhythmia in a real-world setting.

**Methods:**

In this retrospective cohort study, we examined the MIMIC-IV database for vasopressor-dependent patients with septic shock and incident tachyarrhythmia. Employing propensity score matching and restricted cubic splines analysis, we compared the mortality of intravenous esmolol and metoprolol in septic shock patients with tachyarrhythmia.

**Results:**

The primary outcome was 28-day ICU mortality; the secondary outcomes were longitudinal hemodynamic indices (heart rate, blood pressure, lactate). Overall, 31.82% (1,494/4,695) cases died by day 28. Metoprolol was used in 44.6% and esmolol in 1.9%. In multivariable Cox models with spline-informed covariate handling, both esmolol and metoprolol were associated with lower 28-day mortality versus no β_1_-blocker (esmolol HR, 0.69; 95% CI, 0.51–0.95; metoprolol HR, 0.28; 95% CI, 0.25–0.32). Results remained directionally consistent after the concurrent exclusion of sinus tachycardia and ventricular fibrillation and in propensity-score–matched comparisons. Longitudinal analyses showed that survivors across all regimens maintained relatively stable SBP/DBP through day 8, whereas esmolol non-survivors exhibited marked SBP/DBP fluctuations with a concurrent lactate surge around day 2; metoprolol was associated with more stable trajectories.

**Conclusion:**

In real-world septic shock with arrhythmia, intravenous β_1_-blockade was associated with a more favorable 28-day survival trajectory, with metoprolol showing a more prominent survival association and more stable blood-pressure/lactate dynamics compared to esmolol.

## 1 Introduction

Sepsis and septic shock remain leading causes of in-hospital death and impose substantial cardiovascular stress characterized by sympathetic overactivation, high circulating catecholamines, and tachyarrhythmias. Observational data link tachycardia to worse outcomes in sepsis, underscoring the clinical relevance of heart rate (HR) control in this setting (Rudd et al., 2020; Levy et al., 2021; Na et al., 2023).

Current sepsis management protocols emphasize early resuscitation with intravenous fluid boluses and vasopressors to achieve hemodynamic targets (Andrews et al., 2017; Moschopoulos et al., 2023). However, these interventions, while crucial, can lead to significant catecholaminergic adverse effects, potentially causing cardiac dysfunction through sympathetic nerve overstimulation. β_1_-blockers have been proposed as a therapeutic strategy to mitigate these deleterious effects and improve outcomes in sepsis (Suzuki et al., 2017; Eraky et al., 2024). Existing evidence from randomized controlled trials (RCTs) and meta-analyses supports the use of ultra-short acting β_1_-blockers, such as esmolol and landiolol, for heart rate control in septic shock (Kakihana et al., 2020; Hasegawa et al., 2021; Huang et al., 2024; Zheng et al., 2025; Rehberg et al., 2024). A recent comprehensive meta-analysis further indicates that β_1_-blocker treatment generally reduces mortality in critically ill adults and appears safe after initial hemodynamic stabilization, even with concurrent vasopressor use (Morelli et al., 2013). However, these highly controlled studies often employ strict inclusion and exclusion criteria, which can limit the generalizability of their findings to the diverse and complex patient populations encountered in routine practice. In particular, patients with septic shock complicated by tachyarrhythmia are frequently older, multimorbid, and variably resuscitated, with ongoing vasopressor dependence, fluctuating lactate and blood-pressure profiles, and dynamic arrhythmia phenotypes—features that are underrepresented or excluded in traditional RCTs. As a result, there remains a critical gap regarding which β_1_-selective regimen, in whom, and under what hemodynamic conditions these agents are most effective and safe in real-world care. Furthermore, while some meta-analyses have explored the impact of pre-morbid β_1_-blocker exposure on sepsis mortality, the optimal choice and specific effects of different β_1_-selective blockers initiated during sepsis shock in this high-risk cohort remain less clear (Sherman et al., 2016; Chu et al., 2024).

To address these gaps, we leverage the large, real-world MIMIC-IV database to study septic shock complicated by tachyarrhythmia. We conducted a retrospective cohort analysis comparing intravenous esmolol and metoprolol with respect to 28-day survival, and we examined associated factors as well as time-course changes in key hemodynamic indices (e.g., lactate clearance and blood-pressure trajectories) under ongoing vasopressor support. By employing advanced statistical methods, including propensity score matching and restricted cubic splines, to account for confounding and to model dose–response/time-varying effects, our study aims to provide generalizable, bedside-relevant evidence on β_1_-selective blockade in this vulnerable population. These findings are intended to inform clinical decision-making (drug selection, timing, and monitoring of hemodynamic trajectories) and to guide the design of future prospective trials in septic shock with arrhythmia.

## 2 Materials and methods

### 2.1 Study design and setting

We conducted a retrospective cohort study based on the MIMIC-IV database (version 1.0) (Johnson et al., 2021). This is a longitudinal, single-center database, including 35,010 patients admitted with sepsis (Defined by sepsis-3 criteria) (Singer et al., 2016) between 2011 and 2019. Patients admitted to the intensive care unit (ICU) who received conventional treatment for sepsis, according to clinical guidelines for the management of sepsis, and who subsequently developed a tachyarrhythmia, were enrolled. Access to the MIMIC-IV database (version 1.0) was granted following the successful completion of the Collaborative Institutional Training Initiative (CITI) examination by author Chen Xiujuan (Certification number 42391572). The establishment of the MIMIC-IV database was originally approved by the Institutional Review Boards of Beth Israel Deaconess Medical Center and the Massachusetts Institute of Technology. For this specific study, the Ethics Review Committee of Guangdong Provincial People’s Hospital reviewed the research protocol and officially waived the requirement for formal ethical approval and informed consent, given that the analysis utilized de-identified data from a publicly accessible database.

### 2.2 Study population

The main inclusion criteria were 1) age ≥20 years, 2) an acute increase in SOFA score by ≥2 points, 3) confirmed or suspected infection treated with antimicrobial agents, 4) administration of vasopressors necessary to maintain mean arterial pressure at 65 mmHg or more for at least 1 h, 5) tachyarrhythmia occurring while vasopressors were ongoing (heart rate ≥100 beats per min (bpm) sustained for ≥10 min) (Kakihana et al., 2020), and 6) a documented cardiac rhythm of atrial flutter, atrial fibrillation, junctional tachycardia, paroxysmal atrial tachycardia, paroxysmal junctional tachycardia, supra ventricular tachycardia, ventricular fibrillation, or sinus tachycardia. Vasopressors included norepinephrine, phenylephrine, epinephrine, vasopressin, dopamine, and dobutamine. These criteria were used to select patients with septic shock complicated by tachyarrhythmia according to the JSSCG 2016 (Kakihana et al., 2020; Nishida et al., 2018). But we did not include the diagnosis as an inclusion criterion because there was no definite time of diagnosis. Exclusion criteria were 1) pregnancy, 2) use of β_1_-blockers other than esmolol and metoprolol, 3) missing rate of variables >30%, 4) mismatched medications and input events, 5) tachyarrhythmia occurred 7 days after admission to ICU. A schematic illustration of the study design is presented in Supplementary Figure S1.

### 2.3 Outcomes and measures

Our primary outcome was 28-day mortality after ICU admission. Secondary outcomes included changes in heart rate, blood pressure, mean arterial pressure (MAP), systolic blood pressure (SBP), and diastolic blood pressure (DBP) from 0 to 10 days after tachyarrhythmia onset.

In our study, we extracted patients’ parameters containing age, gender, body mass index (BMI), the first 24-h SOFA score, Acute Physiology and Chronic Health Evaluation (APACHE) II score (within 24 h of ICU admission) (Knaus et al., 1985), glasgow coma scale (GCS) score, heart rate, MAP, respiratory rate, SBP, DBP, ratio of PaO2 and FiO_2_ (PaO_2_/FiO_2_), PaCO_2_, cardiac function index, cardiac troponin I, lactate (LA), base excess (BE), anion gap, prothrombin time (PT), hematocrit, hemoglobin, urea nitrogen, creatinine, platelet count (PLT), brain natriuretic peptide (BNP), and N-terminal proBNP (NT-proBNP), time from entering ICU to fluid resuscitation, time from entering ICU to onset of tachyarrhythmia, time from onset of tachyarrhythmia to β_1_-selective blockers start, mechanical ventilation use, premorbid (before tachyarrhythmia onset) β_1_-blocker exposure, time to complete fluid resuscitation, route of first β_1_-blockers after tachyarrhythmia, preexisting conditions such as coronary artery disease (CAD), chronic kidney disease (CKD), chronic obstructive pulmonary disease (COPD), hypertension, diabetes mellitus (DM), hyperlipidemia, asthma, myocardial infarction (MI). In this study, types of administration for crystalloids and albumin included normal saline and lactated Ringer’s (LR) solution, while 5% and 25% HSA for colloids. Adequate fluid resuscitation was defined as giving 30 mL/kg of intravenous crystalloids and albumin within 3 h or 6 h before tachyarrhythmia onset. Preexisting conditions were identified using the International Classification of Diseases code 9 or 10.

The code of outcomes and measures data extraction came mainly from Github (https://github.com/MIT-LCP/mimic-iv). Boxplots were used to detect the outliers of continuous variables. All the baseline characteristics were defined as the closest value recorded before the first tachyarrhythmia onset, except for SOFA and APACHE II scorecores. Cardiac function index, cardiac troponin I, TBIL, count, and percentage of neutrophil, BNP, and NT-proBNP were not included in the analysis because the missing proportion was >30%. The missing percentages of variables included in the analysis were presented in Supplementary Table S1.

### 2.4 Statistical analysis

Continuous data were expressed as median (Q1, 25th percentiles; Q3, 75th percentiles) as they were non-normally distributed data, and multiple group comparisons were performed with Kruskal–Wallis tests. Data normality was assessed using the Kolmogorov–Smirnov tests. Categorical data were expressed as absolute numbers and percentages, and multiple group comparisons were performed by the Chi-square tests. Restricted cubic spline (RCS) functions were used to explore the relationship between 28-day mortality and every continuous variable. The number of knots (knots = 3, 4, or 5) of RCS-transformed models was selected using the minimum Akaike information criterion (AIC). RCS also indicated the objective reference values where hazard ratios were equal to 1. Before multivariate analyses, the missing values of variables were imputed using the R package “missForest”, which can successfully handle missing values ranging from 10% to 30%, particularly in datasets including different types of variables (Stekhoven and Buhlmann, 2012). Cox proportional hazards stepwise regression model was used to assess independent predictors of survival, and performance of the prediction model. The model stability was assessed using bootstrap analysis (1,000 bootstrap samples). The concordance index (C-index) and calibration curve were used to evaluate the model’s predictive accuracy. In addition, to reduce potential selection bias and balance the intergroup difference of covariates, propensity score matching (PSM) analysis using the R package “TriMatch” (Bryer, 2013) was performed. Exact matching was performed on four variables to control for their potential impact on outcomes (route of first β_1_-blockers after tachyarrhythmia, mechanical ventilation, adequate fluid resuscitation, and premorbid β_1_-blocker exposure). We used the OneToN method to match all the covariates with a caliper of 0.20. To improve precision without a commensurate increase in bias, a fixed matched ratio of 2:1:2 was set for none:esmolol: metoprolol (Austin, 2010).

A two-tailed *P* value < 0.05 was considered statistically significant. Post hoc testing was done with the Bonferroni correction, and *P*-values were corrected by dividing by the number of comparisons where appropriate. Statistical analyses were performed using R version 4.1.2 (R Foundation for Statistical Computing) and SAS 9.4 (SAS Institute, Inc., Cary, USA).

## 3 Results

### 3.1 Patient characteristics

We included 4,695 patients with septic shock complicated by tachyarrhythmia receiving ongoing vasopressor support. During the 28-day ICU stay, 31.82% (1,494/4,695) of the patients died. Patients were grouped by the first β_1_-selective regimen after tachyarrhythmia onset: no β_1_-blocker (53.44%, n = 2,509), intravenous esmolol (1.92%, n = 90), and intravenous metoprolol (44.64%, n = 2096).

Baseline characteristics differed across groups (Table 1). In the unmatched cohort, the metoprolol group was older with higher BMI, lower cardiovascular SOFA subscores, and lower lactate and anion gap; premorbid β_1_-blocker exposure and cardiovascular comorbidities (e.g., CAD, hypertension, hyperlipidemia) were more frequent than in the other groups. Fluid resuscitation within 3–6 h or ≤3 h was more common in the esmolol and metoprolol groups versus no β_1_-blocker. After matching, most baseline differences were attenuated (Table 1). Among patients with documented β_1_-blocker exposure before the index tachyarrhythmia, the spectrum and distribution of agents are summarized in Supplementary Table S2.

**TABLE 1:** Baseline characteristics of the study patients.

Characteristics, median (IQR) or as shown	Original (unmatched) data				Matched data			
	None (n = 2,509)	Esmolol (n = 90)	Metoprolol (n = 2096)	*P* value^#^	None (n = 101)	Esmolol (n = 59)	Metoprolol (n = 90)	*P* value^#^
Year of admission	2,153 (2133,2173)	2,147 (2131,2172)	2,152 (2,133–2,173)	0.602	2,155 (2131,2175)	2,147 (2131,2172)	2,154 (2134,2180)	0.537
Age, y	60 (49,70)	62 (49,70)	66 (57,75)^a^ ^,^ ^b^	<0.001	63 (51,73)	62 (49,71)	61 (50,71)	0.890
BMI, kg/m^2^	26 (20.9,31.7)	26.4 (23,30.3)	27.6 (23.3,32.3)^a^	<0.001	26.1 (22.6,32.6)	26 (22.2,29.2)	26.6 (22.6,32)	0.747
Sex (male), n (%)	1,438 (57.3)	48 (53.3)	1,341 (64.0)^a^ ^,^ ^b^	<0.001	57 (56.4)	34 (57.6)	57 (63.33)	0.602
SOFA score								
Total score	4 (3,6)	4 (3,7)	4 (2,6)^a^ ^,^ ^b^	<0.001	4 (3,6)	4 (3,6)	4 (3,6)	0.824
Central nervous	0 (0,1)	0 (0,1)	0 (0,1)	0.079	0 (0,0)	0 (0,1)	0 (0,1)	0.432
Respiratory	2 (1,3)	2 (2,3)	2 (2,3)	0.817	2 (2,3)	2 (2,3)	3 (2,3)	0.766
Cardiovascular	4 (3,4)	4 (1,4)	3 (1,4)^a^ ^,^ ^b^	<0.001	4 (3,4)	4 (1,4)	4 (3,4)	0.789
Liver	0 (0,1)	0 (0,1)	0 (0,0)^a^ ^,^ ^b^	<0.001	0 (0,0)	0 (0,1)	0 (0,0)	0.406
Renal	1 (0,2)	1 (0,3)	0 (0,1)^a^ ^,^ ^b^	<0.001	1 (0,2)	1 (0,2)	1 (0,2)	0.891
Coagulation	0 (0,2)	0 (0,2)	0 (0,1)^a^	0.006	1 (0,2)	1 (0,2)	0 (0,1)	0.378
APACHE II score	18 (14,23)	19 (14,22)	17 (14,22)^a^	<0.001	18 (15,21)	19 (15,21)	17.9 (15,21)	0.816
GCS score								
Total score	15 (15,15)	15 (15,15)	15 (15,15)	0.852	15 (15,15)	15 (15,15)	15 (15,15)	0.479
Motor	5 (1,6)	3 (1,5)^a^	5 (1,6)^b^	<0.001	4 (1,5.9)	4 (1,5)	4.3 (1,6)	0.733
Verbal	0 (0,1)	0 (0,0)	0 (0,0)	0.050	0 (0,0)	0 (0,0)	0 (0,0)	0.671
Eye	2 (1,4)	1 (1,3)^a^	3 (1,4)^b^	<0.001	2 (1,3)	1 (1,3)	2 (1,3)	0.521
Unable	1 (0,1)	1 (1,1)	1 (1,1)	0.116	1 (1,1)	1 (1,1)	1 (1,1)	0.682
Heart rate, beats/min	106 (101,117)	106 (101,116)	104 (100,113)^a^	<0.001	105 (100,114)	106 (101,115)	108 (102,120)	0.156
MAP, mmHg	74 (69,83)	78 (71,85)	76 (70,84)^a^	<0.001	76 (70,84)	76 (69,81)	74.1 (70,82)	0.678
Respiratory rate, beats/min	22 (18,27)	22 (18,27)	20 (17,25)^a^	<0.001	20 (18,26)	22 (18,26)	21.5 (18,26)	0.720
SBP, mmHg	107 (96,119)	108 (96,120)	110 (99,123)^a^	<0.001	107 (97,117)	104 (96,119)	104 (94,115)	0.461
DBP, mmHg	57 (50,66)	60.5 (53,69)	58 (51,65)	0.049	58 (52,69)	57 (50,64)	58 (50,66)	0.617
PaO_2_/FiO_2_, mmHg	232 (162.5,325.7)	237.5 (144,325)	244 (182,325)^a^	0.015	225 (164,291.6)	233.3 (167.5,299.2)	219 (184,284)	0.966
PaCO_2_, mmHg	39 (34,45)	36 (32.5,42.5)	40 (35,45)^a^ ^,^ ^b^	0.001	38.7 (35,43)	37 (33,44)	39 (34,46)	0.440
LA, mmol/L	2.2 (1.4,4)	2.2 (1.3,4)	1.9 (1.4,3)^a^	<0.001	2.2 (1.4,3.7)	2 (1.2,3.5)	2 (1.4,3.7)	0.793
BE, mmol/L	−3 (-7,0)	−4 (-7,0)	−1 (-4,0)^a^ ^,^ ^b^	<0.001	−3 (-7,0)	−3 (-7,0)	−2 (-6,0)	0.325
Anion gap, mEq/L	15 (12,19)	15 (13,19)	14 (11,17)^a^ ^,^ ^b^	<0.001	16 (13,19)	15 (13,19)	15 (12,18)	0.463
PT, sec	15.1 (13.3,18.3)	14.6 (12.5,16.7)	14.4 (13.0,16.3)^a^	<0.001	15.4 (13.7,17.4)	15.3 (12.8,17.7)	15 (12.9,16.5)	0.416
Hematocrit, %	29.6 (26.0,34.0)	30.1 (27.2,35.5)	30.0 (26.9,33.8)	0.022	31.9 (27.1,35.4)	29.3 (26.5,33.2)	29.5 (26.5,35.2)	0.341
Hemoglobin, g/dL	9.7 (8.5,11.2)	10 (9,11.7)	10.0 (8.9,11.4)^a^	<0.001	10.5 (8.9,11.7)	9.6 (8.8,11.1)	9.8 (8.6,12)	0.195
Urea nitrogen, mg/dL	23 (14,39)	24 (14,38)	21 (14,34)^a^	<0.001	26.5 (17,40)	26 (14,40)	22 (16,38)	0.567
Creatinine, mg/dL	1.2 (0.8,1.9)	1.3 (0.8,2.0)	1.0 (0.7,1.6)^a^	<0.001	1.4 (0.9,2.0)	1.3 (0.9,2.2)	1.3 (0.8,2.2)	0.902
PLT, × 10^3^/μL	163 (100,245)	174 (122,268)	170 (121,234)^a^	0.008	157 (101,272)	161 (107,240)	183.5 (114,247)	0.450
Time from entering ICU to fluid resuscitation, h	2.3 (0.8,7.7)	3.0 (0.8,13.8)	2.8 (1,6.9.0)^a^	0.008	2.5 (0.8,6.7)	3 (0.8,11.5)	2.9 (1.4,9.3)	0.442
Time from entering ICU to onset of tachycardia, h	21 (8,51)	26.5 (11,59)	20 (8,48)	0.321	23 (9,62)	29 (11,58)	23 (9,52)	0.824
Time from onset of tachycardia to β1-selective blockers start, h	-	22 (7,66)	33 (12,88)	<0.001	-	30 (11,82)	45.5 (14,105)	0.355
Mechanical ventilation, n (%)	1955 (77.9)	77 (85.6)	1,598 (76.2)	0.068	91 (90.1)	53 (89.8)	80 (88.89)	0.961
Premorbid β-blocker exposure, n (%)	528 (21.0)	37 (41.1)^a^	968 (46.2)^a^	<0.001	35 (34.6)	22 (37.3)	36 (40)	0.747
Adequate fluid resuscitation, n (%)				<0.001				0.975
No	596 (23.8)	28 (31.1)	402 (19.2)^a^ ^,^ ^b^		23 (22.8)	13 (22.0)	17 (18.89)	
3.1–6 h	348 (13.9)	9 (10.0)	193 (9.2)		8 (7.9)	5 (8.5)	8 (8.89)	
Within 3 h	1,565 (62.4)	53 (58.9)	1,501 (71.6)		70 (69.3)	41 (69.5)	65 (72.22)	
Fluid input, 6 h after fluid resuscitation, mL/kg	55.5 (31.0,98.1)	58.4 (26.9,97.5)	36.7 (36.7,129.4)^a^	<0.001	63.9 (31.6,103.1)	62.2 (36.3,97.9)	69.3 (35.8,118.8)	0.720
Route of first β blockers after tachycardia, n (%)				<0.001				-
Intravenous	-	90 (100.0)	1,306 (62.3)^b^		101 (100.0)	59 (100.0)	90 (100.0)	
Oral	-	0 (0.0)	790 (37.7)		0 (0.00)	0 (0.00)	0 (0.00)	
Preexisting conditions, n (%)								
CAD	503 (20.1)	18 (20.0)	883 (42.1)^a^ ^,^ ^b^	<0.001	25 (24.8)	12 (20.3)	15 (16.67)	0.387
CKD	478 (19.1)	19 (21.1)	423 (20.2)	0.589	21 (20.8)	14 (23.7)	16 (17.78)	0.673
COPD	135 (5.4)	8 (8.9)	97 (4.6)	0.133	11 (10.9)	5 (8.5)	5 (5.56)	0.415
Hypertension	1,060 (42.3)	36 (40.0)	1,092 (52.1)^a^ ^,^ ^b^	<0.001	41 (40.6)	22 (37.3)	36 (40)	0.914
DM	638 (25.4)	19 (21.1)	615 (29.3)^a^	0.005	28 (27.7)	14 (23.7)	16 (17.78)	0.265
Hyperlipidemia	662 (26.4)	24 (26.7)	875 (41.8)^a^ ^,^ ^b^	<0.001	28 (27.7)	17 (28.8)	23 (25.56)	0.898
Asthma	219 (8.7)	6 (6.7)	170 (8.1)	0.628	8 (7.9)	2 (3.4)	7 (7.78)	0.492
MI	370 (14.7)	16 (17.8)	433 (20.7)	<0.001	20 (19.8)	10 (16.9)	14 (15.56)	0.736
Outcomes								
Length of stay in ICU, days	5.8 (2.9,10.9)	9.4 (4.6,16.8)^a^	8.6 (4.2,15.3)^a^	<0.001	5.4 (3.6,11)	10.8 (4.8,18.1)	10.8 (6.7,19.6)^a^ ^,^ ^b^	<0.001
Length of stay in hospital, days	12.4 (5.8,22.8)	15.9 (6.6,27.1)	16 (9.2,27.5)^a^	<0.001	12.5 (6.2,23.4)	18.3 (6.8,29.0)	17.8 (11.7,27.7^a^	0.003
28-day mortality, n (%)	1,122 (44.7)	41 (45.6)	331 (15.8)^a^ ^,^ ^b^	<0.001	52 (51.5)	26 (44.1)	23 (25.56)ab	0.001
In-hospital mortality, n (%)	1,162 (46.3)	42 (46.7)	380 (18.1)^a^ ^,^ ^b^	<0.001	52 (51.5)	27 (45.8)	24 (26.67)ab	0.002

Abbreviation: BMI, body mass index; SOFA score, sequential organ failure assessment score; APACHE II score, acute physiology and chronic health evaluation score; GCS score, glasgow coma scale score; MAP, mean arterial pressure; SBP, systolic blood pressure; DBP, diastolic blood pressure; PaO2/FiO2, ratio of PaO2 and FiO2; LA, lactate acid; BE, base excess; PT, prothrombin time; PLT, platelet count; ICU, intensive care units; CAD, coronary artery disease; CKD, chronic kidney disease; COPD, chronic obstructive pulmonary disease; DM, hypertension, diabetes mellitus; MI, myocardial infarction.

^a^ No β-blocker was the referecnce group, *Adj. P* < 0.05.

^b^ Esmolol was the referecnce group, *Adj. P* < 0.05.

^#^ *P* value for the *χ* ^2^ test.

### 3.2 Association between continuous factors with 28-day ICU mortality

**FIGURE 1:**
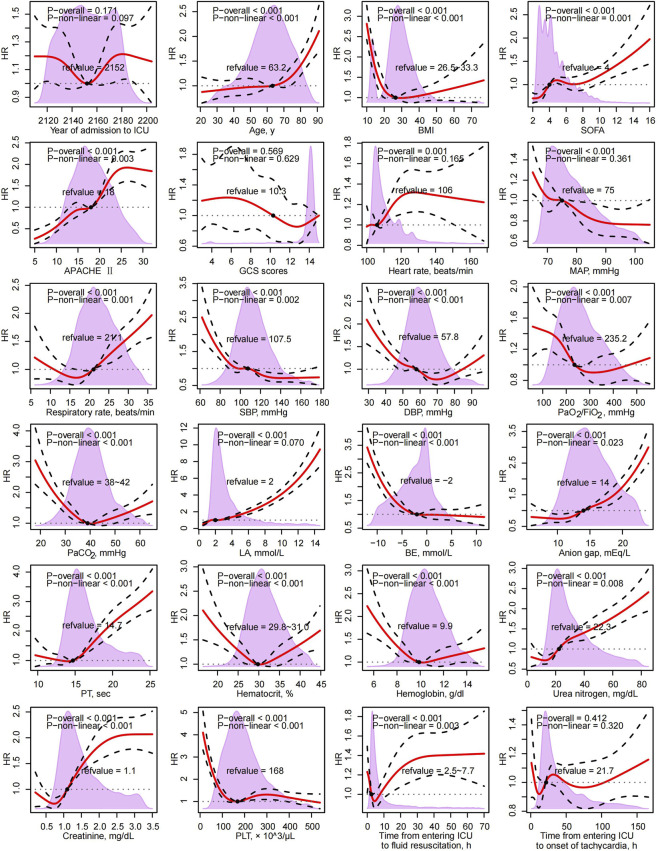
Associations of Continuous factors with 28-Day ICU Mortality. Abbreviation: HR, Hazard Ratio; ICU, Intensive Care Unit; BMI, Body Mass Index; SOFA, Sequential (Sepsis-related) Organ Failure Assessment; APACHE II, Acute Physiology and Chronic Health Evaluation II; GCS, Glasgow Coma Scale; MAP, Mean Arterial Pressure; SBP, Systolic Blood Pressure; DBP, Diastolic Blood Pressure; PaO_2_/FiO_2_, Ratio of Arterial Oxygen Partial Pressure to Fraction of Inspired Oxygen; PaCO_2_, Arterial Partial Pressure of Carbon Dioxide; LA, Lactate; BE, Base Excess; PT, Prothrombin Time; PLT, Platelet Count.

Using restricted cubic splines, year of admission, GCS total, and time from ICU entry to tachyarrhythmia showed no significant linear or non-linear association with 28-day ICU mortality, whereas 21 other continuous variables demonstrated significant linear or non-linear relationships. The observed patterns were approximately linear, J-shaped, U-shaped, or negative within specific ranges (Figure 1).

### 3.3 Determinants of 28-day ICU mortality

For clarity, continuous covariates were dichotomized at RCS-derived reference values before multivariable modeling. In the full cohort, both esmolol and metoprolol were associated with lower 28-day ICU mortality versus no β_1_-blocker (HR 0.69, 95% CI 0.51–0.95; and HR 0.28, 95% CI 0.25–0.32, respectively). Because sinus tachycardia and ventricular fibrillation may represent different clinical entities and could introduce rhythm-related heterogeneity, we performed a sensitivity analysis excluding both categories. Results remained directionally consistent after the concurrent exclusion of sinus tachycardia and ventricular fibrillation (Table 2). Model discrimination was good (C-index 0.77, 95% CI 0.76–0.78). The model demonstrated stable discrimination throughout the follow-up period (Supplementary Figure S2.1). The calibration curve indicated that while the model performed well in the low-to-moderate risk range, it tended to underestimate the actual mortality risk for patients in the highest-risk range (Supplementary Figure S2.2).

**TABLE 2:** Hazard ratio in multiple Cox analysis.

Variable	Category	Overall cohort (n = 4,695)			Cohort excluding sinus tachycardia and ventricular fibrillation (n = 1784)		
		N	Hazard ratio	*P* Value	N	Hazard ratio	*P* Value
Treatment	None	2,509	Reference	​	651	Reference	​
	Esmolol	90	0.69 (0.51,0.95)	0.022	51	0.45 (0.3,0.68)	<0.001
	Metoprolol	2096	0.28 (0.25,0.32)	<0.001	1,082	0.25 (0.21,0.3)	<0.001
Age, y	≤63.2	2,412	Reference	​	1,184	Reference	​
	>63.2	2,283	1.70 (1.52,1.90)	<0.001	600	1.59 (1.34,1.89)	<0.001
Gender	Female	1868	Reference	​	664	Reference	​
	Male	2,827	0.88 (0.79,0.98)	0.004	1,120	0.85 (0.72,1)	0.051
BMI	26.5–33.3	1,437	Reference	​	1,206	Reference	​
	<26.5 or >33.3	3,258	1.41 (1.25,1.58)	<0.001	578	1.59 (1.33,1.9)	<0.001
Cardiovascular SOFA score	Per unit	4,695	1.07 (1.02,1.12)	0.006	1784	1 (0.93,1.08)	0.980
Coagulation SOFA score	Per unit	4,695	1.11 (1.06,1.16)	<0.001	1784	1.07 (0.99,1.14)	0.075
APACHE II	≤18.0	2,444	Reference	​	1,039	Reference	​
	>18.0	2,251	1.35 (1.20,1.52)	<0.001	745	1.33 (1.11,1.6)	0.002
GCS motor	Per unit	4,695	0.94 (0.91,0.98)	0.002	1784	0.95 (0.9,1)	0.070
GCS eye	Per unit	4,695	0.85 (0.79,0.90)	<0.001	1784	0.94 (0.85,1.04)	0.212
Respiratoty rate, beats/min	≤21.0	2,382	Reference	​	884	Reference	​
	>21.0	2,313	1.18 (1.06,1.31)	0.002	900	1.08 (0.92,1.26)	0.376
SBP, mmHg	≤107.5	2,414	Reference	​	892	Reference	​
	>107.5	2,281	0.81 (0.73,0.90)	<0.001	892	0.74 (0.63,0.87)	<0.001
PaO_2_/FiO_2_, mmHg	≤235.2	2,164	Reference	​	890	Reference	​
	>235.2	2,531	0.83 (0.75,0.92)	<0.001	892	0.82 (0.7,0.97)	0.017
LA, mmol/L	≤2.0	2,320	Reference	​	864	Reference	​
	≥2.0	2,375	1.27 (1.13,1.43)	<0.001	920	1.17 (0.98,1.4)	0.078
BE, mmol/L	≤ −2.0	2,559	Reference	​	819	Reference	​
	> −2.0	2,136	0.89 (0.79,1.00)	0.049	965	0.65 (0.54,0.78)	<0.001
Anion gap, mEq/L	≤14.0	2,356	Reference	​	977	Reference	​
	>14.0	2,339	1.20 (1.06,1.35)	0.005	807	1.1 (0.92,1.33)	0.291
Prothrombin time, sec	≤14.7	2,252	Reference	​	1,016	Reference	​
	>14.7	2,443	1.35 (1.21,1.51)	<0.001	768	1.3 (1.09,1.55)	0.003
Blood urea nitrogen, mg/dL	≤22.3	2,351	Reference	​	1,090	Reference	​
	>22.3	2,344	1.21 (1.06,1.38)	0.005	694	1.35 (1.1,1.66)	0.004
Creatinine, mg/dL	≤1.1	2,311	Reference	​	1,067	Reference	​
	>1.1	2,384	1.18 (1.02,1.35)	0.032	717	1.07 (0.87,1.33)	0.518
ICU to fluid resuscitation, h	2.5–7.7	1,219	Reference	​	1,295	Reference	​
	<2.5 or >7.7	3,476	1.14 (1.00,1.29)	0.025	489	1.05 (0.88,1.27)	0.575
Premorbid β-blocker exposure	No	3,162	Reference	​	1,008	Reference	​
	Yes	1,533	1.19 (1.05,1.34)	0.005	776	1.06 (0.9,1.26)	0.467
Adquate fluid resuscitation, h	No	1,026	Reference	​	1,149	Reference	​
	Within 3.0	3,119	0.81 (0.71,0.92)	<0.001	193	0.93 (0.74,1.17)	0.373
	3.1–6.0	550	0.75 (0.63,0.90)	0.001	442	0.92 (0.76,1.11)	0.931
Preexisting CAD	No	3,291	Reference	​	675	Reference	​
	Yes	1,404	0.80 (0.70,0.91)	<0.001	1,109	0.65 (0.54,0.78)	<0.001
Preexisting COPD	No	4,455	Reference	​	116	Reference	​
	Yes	240	1.25 (1.02,1.53)	0.033	1,668	1.31 (0.99,1.73)	0.061

Abbreviation: BMI, body mass index; SOFA, score, Sequential Organ Failure Assessment score; APACHE II, score, acute physiology and chronic health evaluation score; GCS, score, glasgow coma scale score; SBP, systolic blood pressure; LCT, lactate acid; BE, base excess; ICU, intensive care units; CAD, coronary artery disease; COPD, chronic obstructive pulmonary disease.

### 3.4 Survival after propensity score matching

**FIGURE 2:**
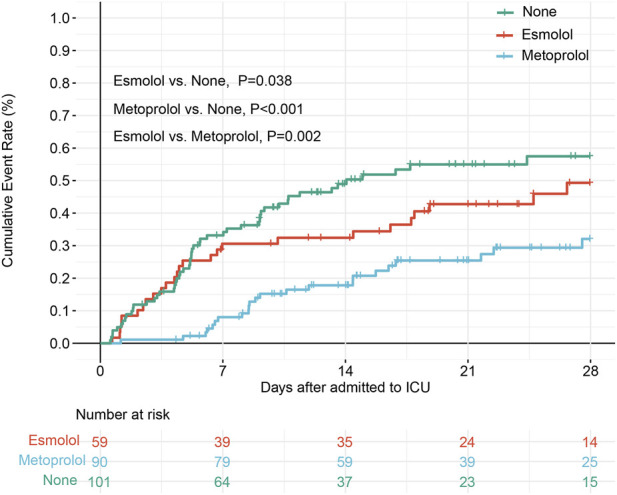
Survival curves of different treatment groups for matched data.

After matching and removing duplicates, 101 no-β_1_-blocker, 59 esmolol, and 90 metoprolol patients remained; 28-day ICU mortality was 51.49%, 44.07%, and 25.56%, respectively. The cumulative 28-day deaths in the metoprolol group remained lower than in the other two groups, with most deaths in the esmolol and no-β_1_ groups occurring within the first 7 days (Figure 2).

### 3.5 Infusions patterns in matched patients

**FIGURE 3:**
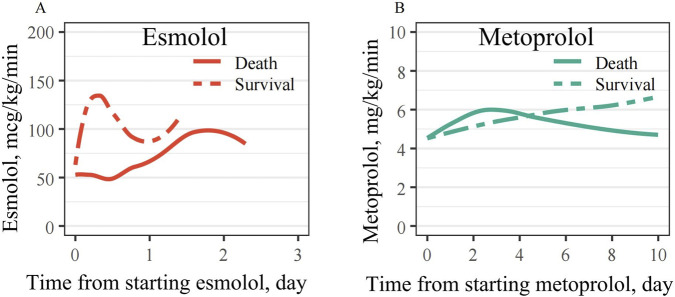
Changes in drug dosage over time. **(A)** Esmolol dose trajectories in survivors and non-survivors. **(B)** Metoprolol dose trajectories in survivors and non-survivors.

Among survivors, esmolol was initiated at higher doses and discontinued within ∼2 days; in non-survivors, higher doses appeared only after ∼2 days (Figure 3A). In the metoprolol group, survivor doses were generally higher than in non-survivors (Figure 3B).

### 3.6 Hemodynamic variables and other outcome data

**FIGURE 4:**
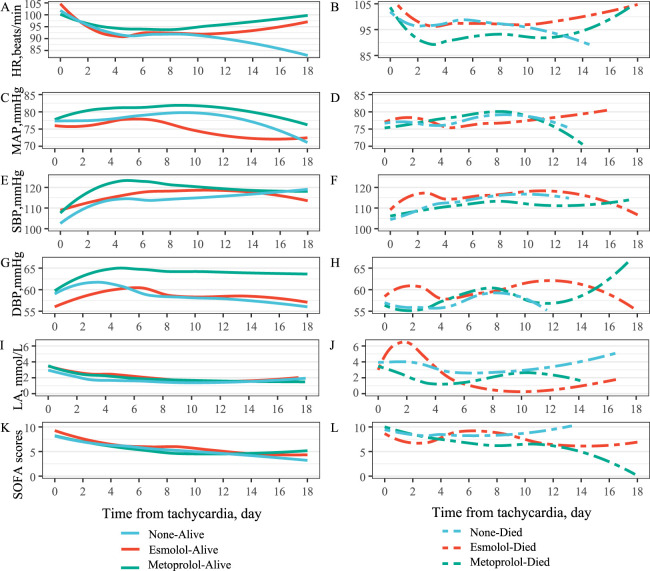
Post-Tachyarrhythmia Trajectories of Hemodynamic and Organ-Dysfunction Indices by β_1_-Blocker Regimen, Stratified by 28-Day Survival (Left: Survivors; Right: Non-Survivors). Abbreviation: HR, Heart rate; MAP, Mean Arterial Pressure; SBP, Systolic Blood Pressure; DBP, Diastolic Blood Pressure; LA, Lactate; SOFA, Sequential (Sepsis-related) Organ Failure Assessment.

Across groups, heart rate declined after tachyarrhythmia and stabilized by day 4; among non-survivors, metoprolol patients exhibited relatively lower HR through day 12. Survivors in all treatment strata maintained relatively stable SBP/DBP through day 8, whereas esmolol non-survivors showed marked SBP/DBP/LA fluctuations around day 2. Lactate and SOFA generally declined over time in survivors (Figures 4A–L). A small subset received both esmolol and metoprolol; mortality and vasopressor-use comparisons for matched patients are provided in Supplementary Table S3, and initial vasopressor types in Supplementary Table S4.

## 4 Discussion

In this real-world cohort study from MIMIC-IV, we examined β_1_-selective blockade in septic shock complicated by arrhythmia—a population largely underrepresented in tightly controlled RCTs and characterized by ongoing vasopressor use and dynamic hemodynamics. β_1_-selective blockers were used in approximately 46.6% of this study population, predominantly metoprolol (44.6%), followed by esmolol (1.9%). Both esmolol and metoprolol were associated with lower 28-day ICU mortality versus no β_1_-blocker, with a greater survival advantage observed for metoprolol. Extending beyond heart-rate control, our longitudinal analyses underscore the importance of hemodynamic trajectories: survivors across all treatment strata maintained relatively stable SBP/DBP through day 8, whereas esmolol non-survivors exhibited marked SBP/DBP fluctuations with a concurrent lactate spike around day 2, suggesting a narrow safety window and the need for vigilant monitoring in vasopressor-dependent shock. These findings complement trial evidence by enhancing generalizability to a heterogeneous ICU population, remain directionally consistent after concurrent exclusion of sinus tachycardia and ventricular fibrillation and following propensity-score matching, and were supported by a multivariable model with good discriminatory performance. Collectively, the results highlight that agent selection, timing of initiation, and continuous appraisal of blood-pressure and lactate dynamics are central to safely translating β_1_-blockade into real-world septic shock care.

Our analysis revealed that patients with higher SOFA scores, lower PaCO2, and a lower proportion of adequate fluid resuscitation were more likely to be included in the non-β_1_-blocker and esmolol groups, indirectly suggesting that these patients were sicker at baseline. Additionally, the esmolol group exhibited lower GCS motor and GCS eye scores compared to the non-β_1_-blocker group. This observed clinical selection bias, where sicker patients might be preferentially treated with esmolol or receive no β_1_-blocker, aligns with the known clinical experience of esmolol’s association with frequent and rapid decreases in heart rate and cardiac output, which clinicians might be hesitant to induce in the most unstable patients (Morelli et al., 2013; Ruggeri et al., 2021). Our use of PSM was specifically designed to mitigate such selection biases inherent in observational data, allowing for more robust comparisons between treatment groups.

Beyond the effects of β_1_-blockers, our study identified several important predictors of 28-day ICU mortality. In univariate analysis, PT showed a J-shaped association, indicating a dramatically increased risk of death when PT exceeded 14.7 s. This aligns with current understanding that coagulation abnormalities are key drivers of organ dysfunction in sepsis and septic shock (Iba et al., 2021; Iba et al., 2023). Our results further confirmed that prolonged PT was associated with a 1.35-fold increased risk of 28-day ICU mortality, even after adjusting for other covariates. Similarly, PaCO_2_ exhibited a U-shaped association, suggesting that both hypercapnia and hypocapnia are detrimental, and maintaining PaCO_2_ within the normal range (38–42 mm Hg) was associated with a lower 28-day ICU death risk. These findings are consistent with observations in patients with cerebral injury (Cai et al., 2022; Robba et al., 2024). The 28-day ICU mortality rate in our cohort of sepsis-related tachyarrhythmia patients was 31.82% (1,494/4,695), which is considerably higher than the 16.14% (5,649/35,010) observed in the overall sepsis cohort within the MIMIC-IV database. This underscores that tachyarrhythmia in sepsis, particularly in vasopressor-dependent patients, is a significant risk factor for worsening prognosis (Kakihana et al., 2020; Magder, 2012). While tachyarrhythmia can serve as a compensatory mechanism for decreased cardiac output in the early, unachieved goal resuscitation phase of septic shock, our study highlights the need for effective management strategies in this high-risk group. We found that adequate fluid resuscitation within 6 h was associated with a decreased risk of 28-day ICU mortality, consistent with previous research (Lee et al., 2014; Lee et al., 2021; Ahlstedt et al., 2024).

A notable finding from our analysis was that patients with premorbid β-blocker exposure (esmolol or metoprolol) had a 19% higher risk of death after adjusting for confounders such as age and SOFA score. This result appears to contradict findings from several systematic reviews and meta-analyses, which generally suggest that premorbid β_1_-blocker exposure is associated with reduced mortality in sepsis. For instance, a pooled analysis of three studies showed an adjusted odds ratio of 0.79 (95% CI: 0.67, 0.92; p = 0.004) for mortality reduction with premorbid β_1_-blocker exposure (Tan et al., 2019). Kuo et al., further suggested that only premorbid β_1_-selective use instead of non-selective β-blocker use was associated with an improvement in 28-day ICU mortality, which was partly attributed to the lower lactate concentrations and a lower percentage of norepinephrine use (Kuo et al., 2021). This discrepancy warrants careful consideration. One potential explanation is residual confounding or “confounding by indication.” Patients with pre-existing conditions requiring chronic β_1_-blocker therapy (e.g., advanced heart failure, severe coronary artery disease, or other significant comorbidities) may represent a sicker population with inherently higher mortality risk, even if their underlying conditions are well-managed. Our study did note that the metoprolol group had higher rates of coronary heart disease and hypertension, as well as higher rates of premorbid β-blocker exposure. While our advanced statistical methods, including PSM, aimed to control for measured confounders, unmeasured or incompletely captured factors related to the severity of underlying disease or the decision to continue/discontinue β_1_-blockers during acute sepsis could contribute to this observed association. Furthermore, the specific context of our cohort—vasopressor-dependent sepsis patients with tachyarrhythmia—might represent a unique, highly unstable subgroup where the physiological response to pre-existing β_1_-blockade differs from broader sepsis populations. Future research should meticulously investigate the interplay between specific types of premorbid β_1_-blockers, the decision to continue or discontinue them during acute sepsis, and patient outcomes in such high-risk cohorts.

The comparison between esmolol and metoprolol is clinically relevant. While esmolol offers superior titratability due to its short half-life, our real-world data suggests that metoprolol was associated with more stable hemodynamic trajectories in this cohort. This may be due to the frequent fluctuations observed in the esmolol group, which could be detrimental in patients with marginal cardiovascular reserve.

Another surprising finding was that CAD emerged as a protective factor in our multivariate analysis. While CAD is generally a risk factor for adverse outcomes in sepsis (Hotchkiss and Karl, 2003; Malomo et al., 2025; Kosyakovsky et al., 2021), our observation might be influenced by the higher prevalence of CAD, hypertension, and premorbid β-blocker exposure in the metoprolol group. It is plausible that patients with pre-existing CAD who received metoprolol, a β_1_-selective blocker, might have benefited from its effects on myocardial oxygen demand and rhythm control, potentially mitigating the expected increase in mortality. Alternatively, this could reflect a form of selection bias where patients with more stable or well-managed CAD were more likely to survive and receive β_1_-blockers in this critical setting. Further investigation, potentially through subgroup analyses based on CAD severity or specific management strategies, is needed to fully elucidate this complex relationship (Kakihana et al., 2020; Raper and Sibbald, 1988).

The predictive performance of our multivariate Cox regression model, with a C-index of 0.77 (95% CI: 0.76–0.78), suggests good accuracy in predicting 28-day ICU death. This is comparable to or better than some existing models in sepsis, though lower than models for specific conditions like candidemia or sepsis shock (Suh et al., 2021; Vallabhajosyula et al., 2018). The calibration curve, however, indicated that our model might underestimate the mortality risk for high-risk patients. This limitation likely stems from residual confounding due to missing key covariates, such as comprehensive cardiac function indices (e.g., ejection fraction, cardiac output), cardiac troponin I, BNP, and NT-proBNP, which had high missing proportions (>30%) and thus could not be included in the analysis. These variables are crucial biomarkers of cardiac dysfunction and overall severity in sepsis, and their absence could lead to an incomplete capture of patient risk. Future studies should prioritize the collection of these critical physiological parameters to enhance the accuracy and generalizability of predictive models in this population.

Our comparative analysis of esmolol and metoprolol is particularly insightful. Both agents were associated with lower 28-day ICU mortality compared to no β_1_-selective blocker use, consistent with a recent meta-analysis showing overall mortality reduction with β_1_-blockers in critically ill adults. However, our finding that the esmolol group had a higher risk of death than the metoprolol group, even after PSM, warrants attention. While esmolol is an ultrashort-acting agent with a rapid half-life and has been shown to be effective for heart rate control (Volz-Zang et al., 1994; Vimala et al., 2022; Maurovich-Horvat et al., 2015), our data suggest that its rapid hemodynamic effects might be challenging to manage in this specific, highly unstable cohort. We observed significant fluctuations in SBP and DBP around day 2 in the esmolol group, potentially linked to rapid decreases in cardiac output. Furthermore, our dose-time curve analysis revealed that survival in the esmolol group was associated with starting at a higher dose and discontinuing within 2 days, whereas non-survivors did not receive high doses until later. Remarkably, commencing doses were significantly different between survival and mortality in the esmolol group. In contrast, metoprolol doses in the survival group were higher than in the mortality group and were maintained for a longer duration, which may be associated with the rapid decreases in cardiac output (Schmittinger et al., 2008). These observations underscore the critical importance of appropriate dosing schemes and vigilant hemodynamic monitoring, especially when using ultra-short-acting agents like esmolol, to ensure optimal patient outcomes and avoid potential harm from rapid hemodynamic shifts.

The study has several limitations. First, as a retrospective analysis, despite the rigorous application of PSM and multiple sensitivity analyses, residual confounding remains possible. Specifically, confounding by indication could not be entirely ruled out, as the clinical choice between esmolol and metoprolol might have been influenced by clinicians' real-time assessment of patient stability or potential for rapid deterioration. For instance, the specific type of tachyarrhythmia (e.g., atrial fibrillation with rapid ventricular response vs. severe sinus tachycardia) was broadly defined, and the clinical decision-making process regarding the initiation or choice of β_1_-blocker, or the decision not to perform cardioversion in vasopressor-dependent patients, could not be fully captured. While our inclusion criteria specified tachyarrhythmia without stopping vasopressors, the absence of explicit data on cardioversion attempts or contraindications in the MIMIC-IV database is a limitation. Future studies should aim to differentiate between various tachyarrhythmia types and capture detailed information on rhythm control strategies. Second, our patient population was selected using sepsis-3 criteria, and while this is a widely accepted definition, it may differ from other analyses or miss atypical presentations. Third, we cannot definitively conclude the extent to which non-cardiac mechanisms of β_1_-selective blockers contributed to the observed mortality improvement, nor whether it was solely due to heart rate reduction. Fourth, as previously discussed, the underestimation of mortality risk in high-risk groups due to missing key cardiac function variables (e.g., cardiac function index, BNP, NT-proBNP) is a notable limitation. Fifth, we did not analyze the effect of the specific type of premorbid β-blockers or the initial vasopressor used after ICU admission on outcomes, which could provide further nuanced insights. Finally, the relatively small sample size of the matched esmolol group (n = 59) significantly limits the statistical power of direct comparisons. Therefore, our findings regarding the comparative effectiveness of these two agents should be considered exploratory and hypothesis-generating rather than definitive, requiring validation in larger, prospective randomized trials.

## 5 Conclusion

In conclusion, this study provides valuable real-world evidence supporting the use of intravenous β_1_-selective blockers in sepsis-related tachyarrhythmia patients requiring vasopressor support. Our findings observed a stronger association between intravenous metoprolol and survival compared to esmolol, particularly in terms of survival and hemodynamic stability. These findings highlight the importance of careful agent selection and suggest that the observed differences in survival may be linked to hemodynamic stability. While acknowledging the limitations inherent in observational studies, our research offers actionable insights for clinical practice and underscores the need for future prospective studies, including well-designed RCTs in specific high-risk subgroups, to further refine optimal management strategies for this challenging patient population.

## Data Availability

Publicly available datasets were analyzed in this study. This data can be found here: https://physionet.org/content/mimiciv/3.1/.
