# Supplementary material for: Survival outcomes, determinants, and hemodynamic trajectories with intravenous β1-selective blockade in septic shock complicated by tachyarrhythmia: a real-world MIMIC-IV cohort study
Source: Front Pharmacol. 2026 May 22;17:1741601. doi: 10.3389/fphar.2026.1741601 (PMC13236955; doi:10.3389/fphar.2026.1741601)
Supplement: Supplementary file 1 [file Supplementaryfile1.docx]

**Supplementary Figure S1**

**
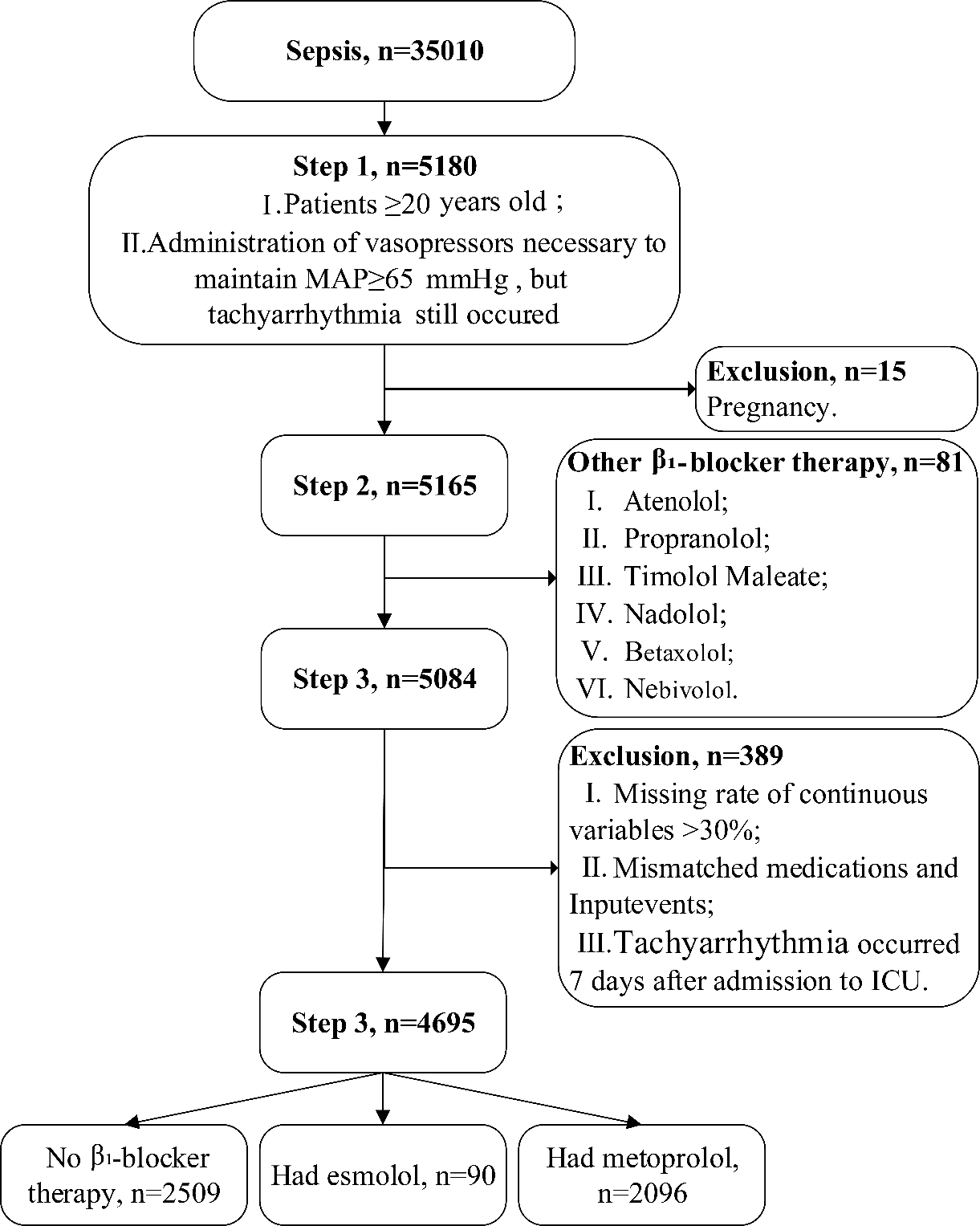
**

**Supplementary Figure S2.1**

Supplementary Figure S2.1. Time-dependent discrimination of the model. This plot displays the concordance index (C-index) over time, reflecting the model's ability to consistently distinguish between survivors and non-survivors throughout the follow-up duration.

**
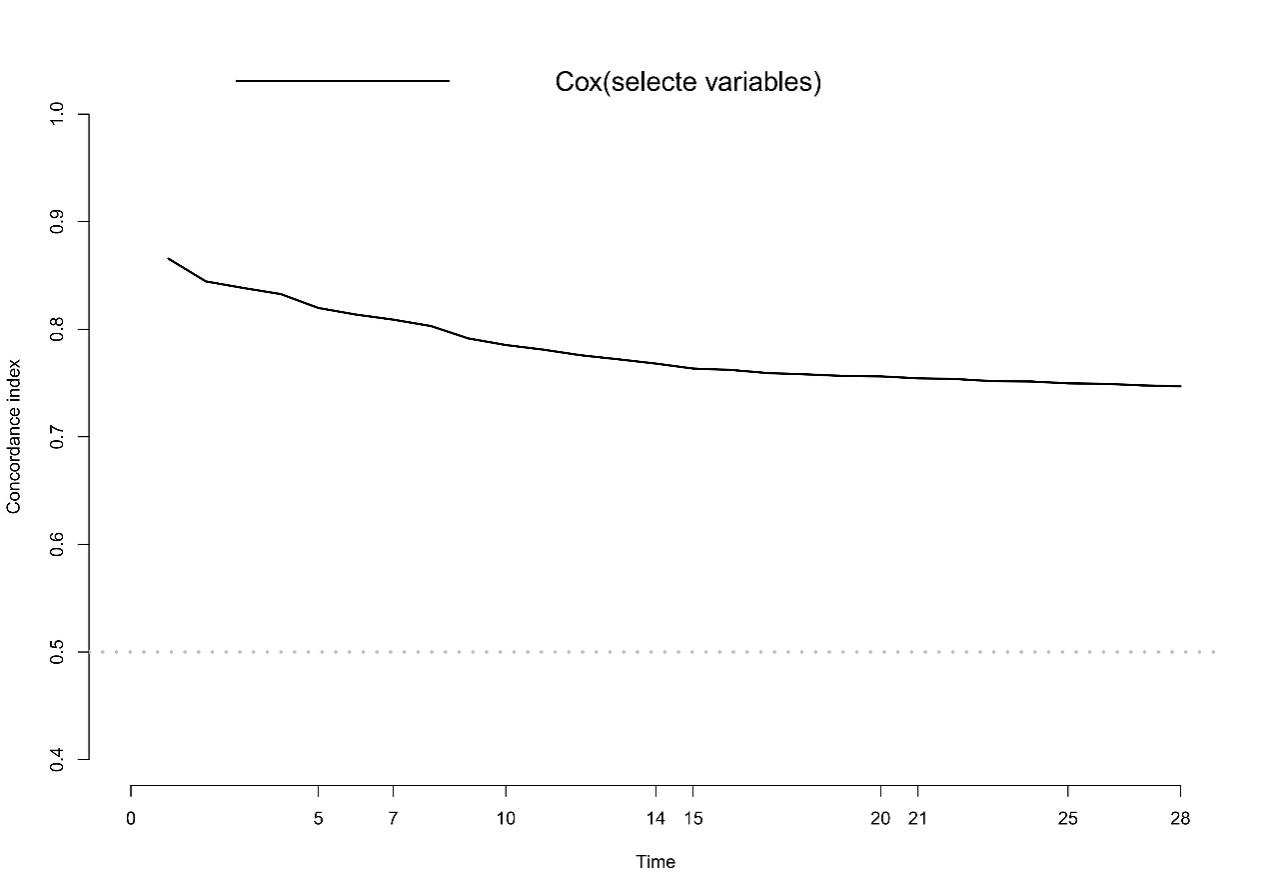
**

**Supplementary Figure S2.2**

Supplementary Figure S2.2. Calibration curve of the model. The plot compares the predicted event probability (x-axis) with the observed event frequency (y-axis). The alignment of the curve with the diagonal suggests good overall calibration, with a slight underestimation observed in the highest-risk patients.

**
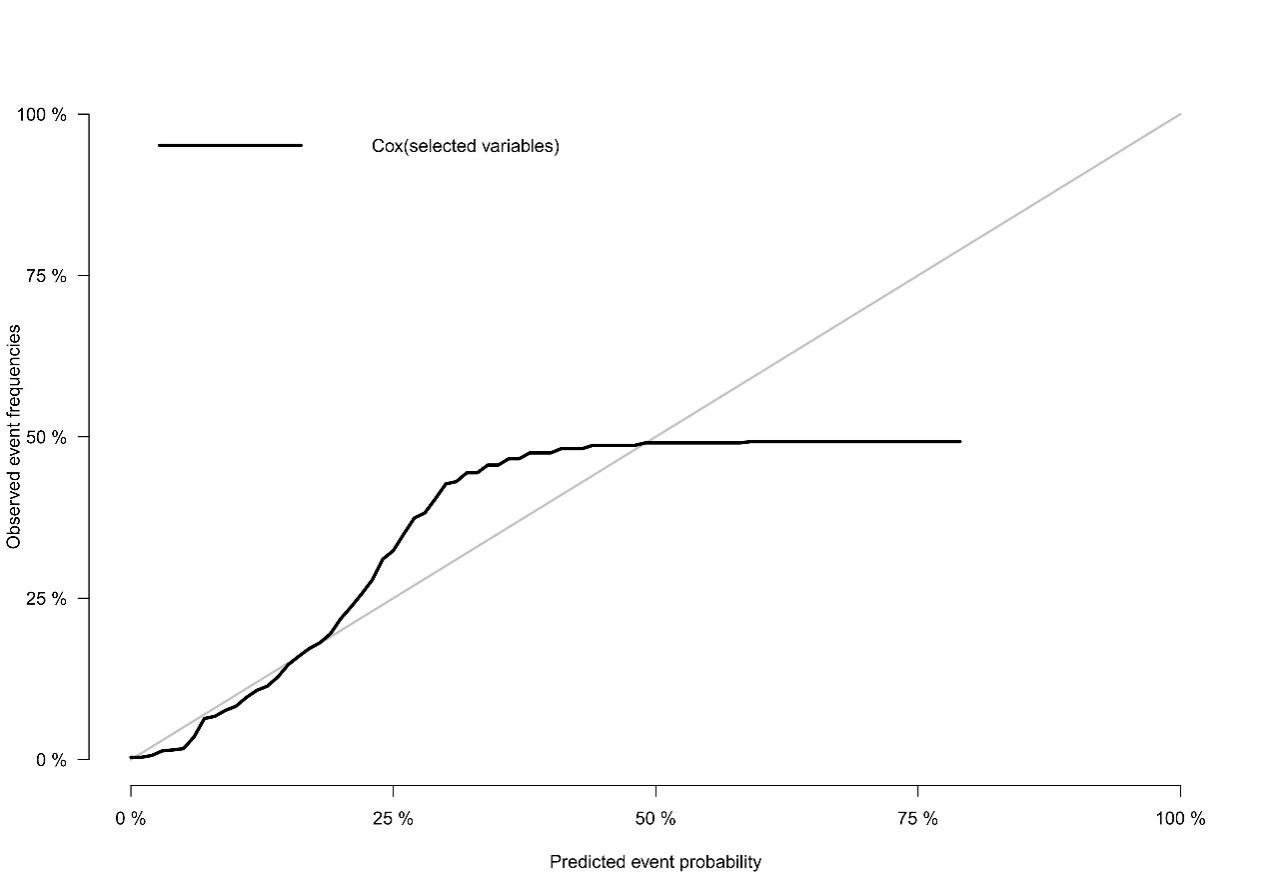
**

**Supplementary Material**

1. **Supplementary Tables**

**1.1 Supplementary Table S1.** Percentage of missing values in continuous variables^*^

| Variables | Percentage (%) |
| --- | --- |
| Year of admission to ICU | 0.0 |
| Age, y | 0.0 |
| BMI | 0.0 |
| Sex (Male), n(%) | 0.0 |
| SOFA scores |  |
| Total SOFA scores | 0.0 |
| Central nervous | 0.0 |
| Respiratory | 0.0 |
| Cardiovascular | 0.0 |
| Liver | 0.0 |
| Renal | 0.0 |
| Coagulation | 0.0 |
| APACHE Ⅱ | 29.5 |
| GCS scores |  |
| Total GCS scores | 1.2 |
| Motor | 2.5 |
| Verbal | 2.0 |
| Eye | 1.7 |
| Unable | 1.2 |
| Heart rate, beats/min | 0.0 |
| Mean arterial pressure, mmHg | 1.4 |
| Respiratory rate, beats/min | 0.0 |
| SBP, mmHg | 0.0 |
| DBP, mmHg | 0.0 |
| PaO_2_/FiO_2_, mmHg | 21.6 |
| PaCO_2_, mmHg | 3.1 |
| Lactate acid, mmol/L | 4.8 |
| Base excess, mmol/L | 5.6 |
| Anion gap, mEq/L | 7.6 |
| Prothrombin time, sec | 11.6 |
| Hematocrit, % | 3.2 |
| Hemoglobin, g/dl | 2.9 |
| Urea nitrogen, mg/dL | 3.4 |
| Creatinine, mg/dL | 8.3 |
| Platelet count, × 10^3^/μL | 2.0 |
| Time from entering ICU to fluid resuscitation, h | 0.0 |
| Time from entering ICU to onset of tachyarrhythmia, h | 0.0 |

*Enumeration data such as mechanical ventilation and premorbid β-blocker exposure were obtained through medical orders or diagnostic records (patients without these records have been excluded), and there was no missing.

1.2 **Supplementary Table S2.** Premorbid β-blocker exposure

| Type of β-blocker before tachyarrhythmia | Route | Original (Unmatched) Data | | | |  | Matched Data | | | |
| --- | --- | --- | --- | --- | --- | --- | --- | --- | --- | --- |
|  |  | Total (n=1533) | None (n=528) | Esmolol (n=37) | Metoprolol (n=968) |  | Total (n=93) | None (n=35) | Esmolol (n=22) | Metoprolol (n=36) |
| Esmolol | Intravenous | 38(2.48) | 10(1.89) | 8(21.62) | 20(2.07) |  | 6(6.45) | 0(0.00) | 4(18.18) | 2(5.56) |
| Atenolol | Oral | 63(4.11) | 21(3.98) | 0(0.00) | 42(4.34) |  | 2(2.15) | 1(2.86) | 0(0) | 1(2.78) |
| Metoprolol | Intravenous | 520(33.92) | 163(30.87) | 15(40.54) | 342(35.33) |  | 43(46.24) | 16(45.71) | 10(45.45) | 17(47.22) |
| Metoprolol | Oral | 856(55.84) | 289(54.73) | 14(37.84) | 553(57.13) |  | 40(43.01) | 16(45.71) | 8(36.36) | 16(44.44) |
| Nadolol | Oral | 41(2.67) | 34(6.44) | 0(0.00) | 7(0.72) |  | 2(2.15) | 2(5.71) | 0(0.00) | 0(0.00) |
| Propranolol | Oral | 15(0.98) | 11(2.08) | 0(0.00) | 4(0.41) |  | 0(0.00) | 0(0.00) | 0(0.00) | 0(0.00) |

**1.3 Supplementary Table S3**

Mortality and vasopressor use comparison based on the presence or absence of drug combination in matched patients

| Group | Subgroup | Number of patients* | 28-day ICU Death, n (%) | Vasopressor use, Median (Q1, Q3) days^*^ |
| --- | --- | --- | --- | --- |
| None | None | 101 | 52(51.49) | 2.4(1.1-5.5) |
| Esmolol | Esmolol | 41 | 21(51.22) | 3.4(1.4,9.9) |
|  | Esmolol+ Metoprolol | 18 | 5(27.78) | 6.9(3.8,16.6) |
| Metoprolol | Metoprolol | 87 | 21(24.14) | 3.1(1.2,10.1) |
|  | Metoprolol+esmolol | 3 | 2(66.67) | 12.0(0.3-18.0) |

^*^T0: The time of tachyarrhythmia.

1.4 **Supplementary Table S4.** Type of first vasopressor used after admission to the ICU

| Type of vasopressor | Original (Unmatched) Data | | | |  | Matched Data | | | |
| --- | --- | --- | --- | --- | --- | --- | --- | --- | --- |
|  | Total (n=4569) | None (n=2509) | Esmolol (n=90) | Metoprolol (n=2096) |  | Total (n=250) | None (n=101) | Esmolol (n=59) | Metoprolol (n=90) |
| Dobutamine | 47(1.00) | 29(1.16) | 1(1.11) | 17(0.81) |  | 2(0.80) | 1(0.99) | 1(1.69) | 0(0.00) |
| Dopamine | 185(3.94) | 108(4.3) | 2(2.22) | 75(3.58) |  | 7(2.80) | 4(3.96) | 2(3.39) | 1(1.11) |
| Epinephrine | 280(5.96) | 115(4.58) | 6(6.67) | 159(7.59) |  | 11(4.40) | 3(2.97) | 2(3.39) | 6(6.67) |
| Norepinephrine | 2112(44.98) | 1350(53.81) | 39(43.33) | 723(34.49) |  | 122(48.80) | 53(52.48) | 27(45.76) | 42(46.67) |
| Vasopressin | 129(2.75) | 74(2.95) | 6(6.67) | 49(2.34) |  | 13(5.20) | 6(5.94) | 4(6.78) | 3(3.33) |
| Phenylephrine | 1942(41.36) | 833(33.2) | 36(40) | 1073(51.19) |  | 95(38.00) | 34(33.66) | 23(38.98) | 38(42.22) |
